# Subretinal fluid in macular edema secondary to branch retinal vein occlusion

**DOI:** 10.1038/s41598-024-64047-y

**Published:** 2024-06-13

**Authors:** Donghyun Jee, Soyoung Park, Jin-woo Kwon

**Affiliations:** Department of Ophthalmology and Visual Science, St. Vincent’s Hospital, College of Medicine, the Catholic University of Korea, Seoul, Korea

**Keywords:** Predictive markers, Prognostic markers, Retinal diseases

## Abstract

We identified characteristics of patients with subretinal fluid (SRF) in macular edema (ME) secondary to branch retinal vein occlusion (BRVO) and determined their clinical outcomes after anti-vascular endothelial growth factor (VEGF) treatment. Fifty-seven eyes of BRVO patients with ME were divided into two groups according to the presence or absence of SRF at diagnosis. We compared the aqueous profiles, ocular and systemic characteristics at baseline, and the clinical outcomes. The SRF group had significantly greater central subfield thickness (CST) values and poorer best-corrected visual acuity (BCVA) at baseline compared to the non-SRF group. The former group had significantly higher aqueous levels of interleukin-8, VEGF, and placental growth factor. CST reduction and BCVA improvement during treatment were significantly greater in the SRF group than in the non-SRF group. Consequently, CST values were significantly lower in the SRF group than in the non-SRF group at 12 months, when BCVA did not differ significantly between the two groups. The SRF group required more frequent anti-VEGF treatment over 12 months and exhibited a higher rate of macular atrophy. Based on the aqueous profiles and the number of treatments required, the presence of SRF in BRVO patients appears to be associated with higher disease activity.

## Introduction

Retinal vein occlusion (RVO) is the second most common retinal vascular disorder worldwide; an estimated 28.06 million people lived with RVO in 2015^1^. It can cause permanent visual disturbance attributable to macular ischemia triggered by RVO itself and/or its associated complications, which include macular edema (ME), neovascular glaucoma, and vitreous hemorrhage^2^. As treatment modalities have improved, early diagnosis and appropriate treatment of complications are required to enhance visual function and ensure good clinical results^3^.

RVO is divided into branch RVO (BRVO) and central RVO (CRVO) depending on the obstruction site. BRVO is far more prevalent than CRVO^4^. ME is the most frequent cause of visual disturbance in patients with BRVO; inflammatory cells, cytokines, growth factors, and enzymes contribute to the condition^5–7^. Anti-vascular endothelial growth factor (VEGF) and steroid implants have been used to treat ME, and have yielded good clinical results in terms of anatomical and visual outcomes^8,9^. However, ME recurrence is common in patients with BRVO, who therefore usually require regular examinations and repeated treatment. Optical coherence tomography (OCT) and OCT angiography (OCTA) data, measurements of biomarker levels in ocular fluid, and systemic evaluation have been used to predict prognoses and the responses to customized treatments^10–12^. The ME components evident in OCT consist of intraretinal fluid and subretinal fluid (SRF). The effects of each fluid type on prognosis have been studied intensively in patients with diabetic ME (DME) and age-related macular degeneration, and good progress has been made^13–16^. However, few studies have explored the effects of SRF on BRVO prognosis. Although several groups have explored the mechanism of SRF development, no consensus has been attained^17–19^. In this study, we explored the characteristics and prognoses of patients with SRF in ME secondary to BRVO.

## Results

We enrolled 57 BRVO with ME eyes of 57 patients of mean age 65.54 ± 11.69 years (19 males and 38 females). At baseline, the mean best-corrected visual acuity (BCVA, log MAR) was 0.56 ± 0.34 and the mean central subfield thickness (CST) was 460.54 ± 149.23 µm. Thirty-eight patients (66.67%) had major BRVO and 19 (33.33%) had macular BRVO; twenty-five (43.86%) patients evidenced SRF in ME.

Table 1 summarizes their systemic and ocular characteristics, classified according to the presence or absence of SRF. The SRF group had significantly greater CST values and poorer BCVA compared to the non-SRF group (540.68 ± 158.96 µm vs. 397.94 ± 106.96 µm P < 0.001 and 0.69 ± 0.36 vs. 0.45 ± 0.28, P = 0.010, respectively). Levels of aqueous IL-8, VEGF, and PlGF were significantly higher in the SRF group (71.56 ± 119.40 pg/mL vs. 29.07 ± 29.12 pg/mL, P = 0.013, 102.27 ± 98.77 pg/mL vs. 54.03 ± 51.94 pg/mL, P = 0.007, and 4.62 ± 2.87 pg/mL vs. 2.81 ± 1.34 pg/mL, P = 0.007, respectively).

**Table 1:** Demographics and clinical characteristics of BRVO patients by SRF status at baseline.

	No SRF group (n = 32)	SRF group (n = 25)	*P*
Systemic factors			
Sex (male: female)	9:23	10:15	0.345
Age (years)	65.50 [57.00; 76.50]	61.00 [58.00; 71.00]	0.425
Diabetes mellitus	10 (31.25%)	8 (32.00%)	0.952
Hypertension	20 (62.50%)	13 (52.00%)	0.426
Dyslipidemia	8 (25.00%)	7 (28.00%)	0.799
Aqueous humor cytokines			
IL-1β (pg/mL)	0.00 [0.00; 0.00]	0.00 [0.00; 0.00]	0.225
IL-2 (pg/mL)	0.20 [0.00; 4.50]	2.24 [0.00; 10.96]	0.112
IL-6 (pg/mL)	5.52 [3.01; 11.07]	8.45 [3.87; 14.63]	0.115
IL-8 (pg/mL)	19.21 [7.43; 42.36]	35.76 [22.15; 73.33]	0.013
IL-10 (pg/mL)	0.53 [0.18; 1.13]	0.84 [0.28; 1.14]	0.290
IL-17 (pg/mL)	0.00 [0.00; 1.21]	0.00 [0.00; 2.16]	0.263
TNF-α (pg/mL)	3.48 [1.87; 5.17]	4.79 [3.19; 6.38]	0.071
VEGF (pg/mL)	40.37 [27.77; 77.20]	64.23 [47.50; 114.66]	0.007
PlGF (pg/mL)	2.75 [1.86; 3.62]	3.76 [2.81; 4.86]	0.007
Ocular factor			
BCVA (log MAR)	0.40 [0.20; 0.70]	0.70 [0.40; 1.00]	0.010
Major BRVO	18 (56.25%)	20 (80.00%)	0.059
OCT findings			
Baseline CST (µm)	355.00 [324.00; 445.50]	510.00 [406.00; 685.00]	< 0.001
EZD grade			
0	21 (65.62%)	10 (40.00%)	0.096
1	6 (18.75%)	11 (44.00%)	
2	5 (15.62%)	4 (16.00%)	

Values are expressed as the mean ± SD or median and interquartile range, as appropriate.

*BRVO* branch retinal vein occlusion, *SRF* subretinal fluid, *IL* interleukin, *TNF* tumor necrosis factor, *VEGF* vascular endothelial growth factor, *PlGF* placental growth factor, *BCVA* best-corrected visual acuity, *CST* central subfield thickness, *EZD* ellipsoid zone disruption.

In terms of the clinical outcomes of the enrolled patients at 12 months, the average CST fell to 258.96 ± 32.35 µm and the logMAR BCVA improved to 0.38 ± 0.29.

The clinical outcomes of the SRF and non-SRF groups at 12 months are summarized and compared in Table 2. The SRF group achieved significantly greater CST reduction compared to the non-SRF group (298.12 ± 145.04 µm vs. 126.16 ± 108.30 µm, P < 0.001); the CST values of the SRF group were significantly lower than those of the non-SRF group at the 12 month check-up (242.56 ± 37.62 µm vs. 271.78 ± 20.20 µm, P = 0.001). Four patients, all from the SRF group, had CSTs < 200 µm (P = 0.019). BCVA improvement was also significantly greater in the SRF group (− 0.12 ± 0.20 vs. − 0.25 ± 0.25, P = 0.039); there was no significant difference in BCVA values between the two groups at the 12-month check-up (P = 0.340). However, the SRF group required more treatments (3.56 ± 1.42 vs. 2.72 ± 1.14, P = 0.040).

**Table 2:** Outcomes of BRVO treatment by SRF status.

	No SRF group (n = 32)	SRF group (n = 25)	*P*
OCT findings			
CST (µm)			
At baseline	355.00 [324.00; 445.50]	510.00 [406.00; 685.00]	< 0.001
At 12 months	277.50 [262.50; 285.00]	249.00 [221.00; 272.00]	0.001
Reduction	88.50 [49.50; 172.50]	287.00 [172.00; 407.00]	< 0.001
≤ 200 µm at 12 months	0 (0.00%)	4 (16.00%)	0.019
EZD grade at 12 months			
0	24 (75.00%)	13 (52.00%)	0.169
1	5 (15.62%)	6 (24.00%)	
2	3 (9.38%)	6 (24.00%)	
BCVA (LogMAR)			
At baseline	0.40 [0.20; 0.70]	0.70 [0.40; 1.00]	0.010
≤ 0.3 at baseline	15 (46.88%)	6 (24.00%)	0.076
> 0.5 at baseline	9 (28.12%)	15 (60.00%)	0.016
At 12 months	0.30 [0.10; 0.40]	0.30 [0.30; 0.50]	0.340
≤ 0.3 at 12 months	19 (59.38%)	14 (56.00%)	0.798
> 0.5 at 12 months	5 (15.62%)	5 (20.00%)	0.667
improvement	− 0.10 [− 0.20; 0.00]	− 0.20 [− 0.50; 0.00]	0.039
Required doses of IVB in 12 months	3.00 [2.00; 4.00]	3.00 [3.00; 5.00]	0.040

Values are expressed as the mean ± SD or median with interquartile ranges, as appropriate.

*BRVO* branch retinal vein occlusion, *SRF* subretinal fluid, *CST* central subfield thickness, *BCVA* best-corrected visual acuity, *IVB* intravitreal bevacizumab.

**Figure 1:**
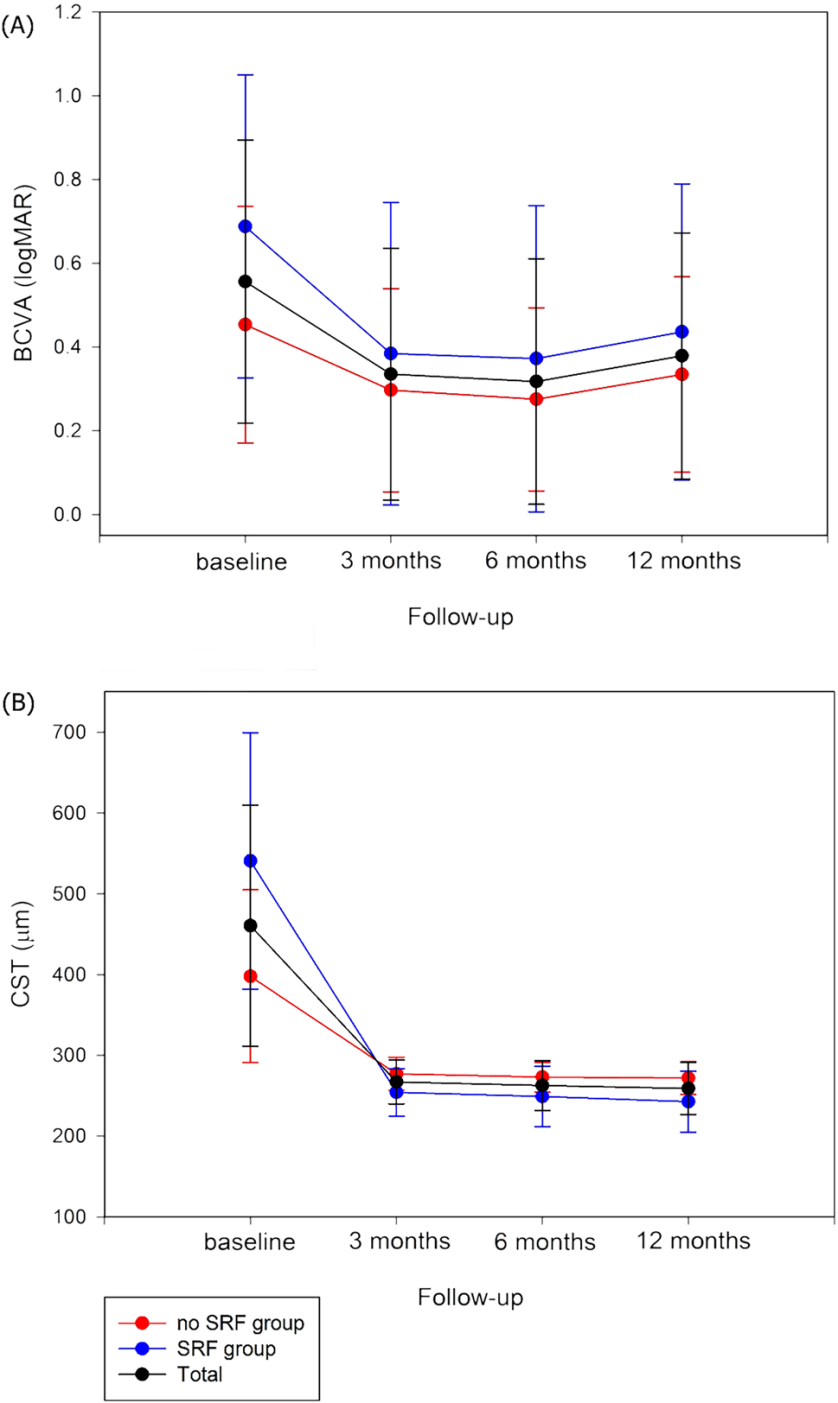
(**A**) Mean logMAR BCVA changes from baseline to month 12 in patients with and without SRF in ME secondary to BRVO. (**B**) Mean CST changes from baseline to month 12 in patients with and without SRF. *BCVA* best-corrected visual acuity, *SRF* subretinal fluid, *ME* macular edema, *BRVO* branch retinal vein occlusion, *CST* central subfield thickness.

Figure 1 shows the changes in BCVA and CST values. Mean CST values did not change noticeably after 3 months, but mean BCVA values in both groups were optimal at 6 months and slightly poorer at 12 months.

## Discussion

We designed this study to determine the characteristics of patients who had SRF in ME secondary to BRVO and its effects on visual function at baseline and clinical outcomes. This is the first study to identify the visual prognosis of BRVO patients with SRF and to find that levels of inflammatory cytokines and VEGFs are elevated in the aqueous humor of such patients.

Although the SRF group exhibited significantly higher CST values and poorer BCVA at baseline, the reduction in CST was greater and the improvement in BCVA was better than in the non-SRF group over 12 months of treatment. Thus, CST values decreased significantly more in the former group than in the latter group; there were no significant differences in BCVA between the two groups. However, although the CST values were reversed, BCVA remained better in the group lacking SRF. In other words, the visual outcomes of the SRF group did not correspond to the CST results. We infer that this result could be associated with more high grades of ellipsoid-zone (EZ) disruption at baseline, more macular atrophy, and more frequent ME recurrence in the SRF group.

SRF is a common finding not only in RVO but also in neovascular age-related macular degeneration, DME, and central serous chorioretinopathy^20^. VEGF mediates dysfunction of the RPE and outer retinal barrier and subsequent fluid leakage into the subretinal space^21,22^. Considering the good responsiveness of SRF to anti-VEGF, VEGF appears to play a crucial role. However, the different responsiveness for each disease indicates that there are other factors, such as inflammation, mediating the development of SRF^20^. In terms of BRVO, previous studies have suggested that SRF also originates from the movement of intraretinal fluid secondary to vascular leakage from congested retinal veins outside the macular area^17,23^. Studies on human vitreous or aqueous samples have suggested that excessive vascular permeability that develops after VEGF upregulation contributes to the appearance of SRF in patients with BRVO^19,24^. Although increased aqueous and vitreous levels of VEGF and IL-6 have been reported in CRVO patients^18^, our study provides the first evidence that both VEGF and IL-8 are associated with SRF development in BRVO patients. Considering these results, it can be inferred that the development of SRF is related not only to VEGF but also to inflammatory factors.

Previously, we reported the characteristics of DME patients with SRF^16^. Such patients evidenced increased levels of certain inflammatory cytokines and VEGFs in aqueous humor compared to others, and had higher rates of macular atrophy, as did the BRVO patients with SRF in the present study. However, in patients with DME, SRF did not affect BCVA at baseline or the number of treatments required. Considering the aqueous profiles and the difference in ME recurrence frequency in BRVO patients with SRF, we suggest that the SRF status of BRVO is a better biomarker of disease activity than is the SRF status of DME. However, further studies are needed to identify the reasons for the different clinical features of SRF in DME and BRVO.

Some recent studies have reported that foveal fluctuation and ME recurrence affect visual outcomes^25–27^. Since we used the PRN regimen, the larger number of treatments required to address ME in the SRF group indicates more frequent ME recurrence and greater CST fluctuation. Although not reported above, we found that the mean between-group logMAR BCVA difference increased from 3 to 12 months, being 0.087 at 3, 0.097 at 6, and 0.102 at 12 months. This may have reflected CST fluctuations, given the more frequent ME recurrence in the SRF group during treatment. However, the differences were not statistically significant.

Our study had certain limitations. Two patients in the no-SRF group and three patients in the SRF group were excluded from this study due to dexamethasone implant treatments. This may mean that patients with high disease activity were excluded. We used frequency of treatments as one of parameter of disease activity. The implant has strong efficacy with long action duration, thus, for a fair comparison, patients who used this option had no choice but to be excluded. The Intravitreal bevacizumab (IVB) treatments used the PRN regimen and the follow-up duration was relatively short. More research is needed to determine whether more potent agents or proactive treatments produce better outcomes. As ME recurrence occurred more in the SRF group than in the non-SRF group, and BCVA values differed between the two groups over time, longer follow-up may yield more meaningful results. In addition, OCTA data should have been considered, but we lacked such data for some patients. Our next study will enroll more patients, and all will undergo OCTA.

In conclusion, based on the aqueous profiles and the number of treatments required, the presence of SRF in BRVO patients appears to reflect higher disease activity in such patients compared to others. Although BRVO patients with SRF had lower BCVA values at baseline, the visual outcomes of the two groups did not differ significantly after treatment.

## Methods

This study adhered to all relevant tenets of the Declaration of Helsinki and was approved by the Institutional Review Board of the Catholic University of Korea (approval number VC21RISI0061). All participants provided written informed consent for the use of their clinical records. We enrolled newly diagnosed and treatment-naïve BRVO patients with ME, a CST ≥ 300 µm, and a follow-up period of at least 12 months. If both eyes met the inclusion criteria, one eye was randomly chosen. The exclusion criteria were ME or hemorrhage attributable to another cause, a history of uveitis, intraocular surgery, and/or laser treatment. We also excluded those with macular diseases that can affect macular thickness; thus, subjects with an epiretinal membrane and/or vitreomacular adhesion or traction. All patients underwent ophthalmic examinations that included measurement of BCVA, fundus assessment, fluorescein angiography, and OCT (Cirrus High-Definition OCT; Carl Zeiss Meditec, Dublin, CA, USA). We selected seven consecutive horizontal B-scans with the scan centered on the fovea, and the three scans immediately above and below. The EZ disruptions in all scans were averaged and graded as 0 (intact), 1 (focal disruption ≤ 250 µm), or 2 (focal disruption > 250 µm)^28^. BRVO was diagnosed by retinal specialists based on typical features evident in fundus examination and/or fluorescein angiography. The BRVO subtypes were classified by the site of vascular occlusion, as described previously^29^. We diagnosed major BRVO when a temporal arcade vein or branch extending to the peripheral retina beyond the retinal vascular arcades was occluded, and macular BRVO when the occlusion was confined to between the superior and inferior retinal temporal vascular arcades^30^.

IVB (Avastin; Genetech, San Francisco, CA, USA) was administered using the pro re nata (PRN) regimen when the CST was ≥ 300 µm at any monthly follow-up. All patients provided written informed consent before receiving IVB and were informed that the use of the drug was off-label. We administered up to three consecutive monthly injections of 1.25 mg IVB if ME was refractory. If these injections failed to resolve ME, we switched to intravitreal dexamethasone and excluded such patients from the study.

We evaluated clinical outcomes at 3, 6, and 12 months. All patients were divided into two groups based on the presence or absence of the SRF component in ME at baseline. We compared their baseline characteristics and clinical outcomes.

### Assessments of cytokines and growth factors

We measured the concentrations of interleukin (IL)-1β, IL-2, IL-6, IL-8, IL-10, IL-17, tumor necrosis factor (TNF)-α, placental growth factor (PlGF), and VEGF in 75-µL samples of aqueous humor. Antibodies against these materials were immobilized on beads; 75-µL aliquots of Calibrator Diluent RD6–52 (R&D Systems, Minneapolis, MN, USA) were added to the samples, followed by addition of the beads and incubation for 2 h, and further incubation for 1 h after addition of the detection antibodies, and a final incubation for 30 min after addition of the streptavidin–phycoerythrin reagent. All samples were analyzed using a Luminex xMAP system (Austin, TX, USA).

### Statistical evaluation

Statistical analyses were performed using IBM SPSS Statistics for Windows version 21.0 (IBM Corp., Armonk, NY, USA). After performing a normality test on all data, a parametric or non-parametric test was performed depending on the results. The Student t-test, Mann–Whitney *U*-test, and chi-square test were used, as appropriate, to compare values or categorical variables of baseline characteristics and clinical outcomes between the patient subgroups. All data in the text are presented as averages ± standard deviations. The statistical significance level was set at P < 0.05 (Supplementary information).

### Supplementary Information

Supplementary Information.

## Data Availability

Data available within the article or its supplementary materials.
